# Effects of CYP3A43 Expression on Cell Proliferation and Migration of Lung Adenocarcinoma and Its Clinical Significance

**DOI:** 10.3390/ijms24010113

**Published:** 2022-12-21

**Authors:** Qi-Yao Wei, Andy T. Y. Lau, Hai-Ying Mo, Qiu-Hua Zhong, Xiao-Yun Zhao, Fei-Yuan Yu, Jin Han, Yu-Yao Wu, Yan-Ming Xu

**Affiliations:** 1 Laboratory of Cancer Biology and Epigenetics, Department of Cell Biology and Genetics, Shantou University Medical College, Shantou 515041, China; 2 Department of Pathology and Medical Biology, University Medical Center Groningen, University of Groningen, 9700 RB Groningen, The Netherlands

**Keywords:** CYP3A43, lung adenocarcinoma, co-expression gene, ERK1/2 signaling

## Abstract

The cytochrome P450s (CYP450s) include key oxidative enzymes involved in the metabolism of various carcinogens and anticancer drugs. Bioinformatic studies have demonstrated the association of CYP3A43 with liver cancer and ovarian cancer. However, the biological function of CYP3A43 in tumor progression remains unclear. To further reveal the role of CYP3A43 in tumor progression, we first analyzed the data from the UALCAN database and found that CYP3A43 was negatively correlated to the cancer staging and lymph node metastasis of lung adenocarcinoma (LUAD). We established stable CYP3A43-knockdown LUAD H1299 cell line and found that its knockdown enhanced cell proliferation, colony formation, and migration in vitro, and promoted the growth of tumor xenograft in vivo. Interestingly, when CYP3A43 was ectopically-expressed in the LUAD cell lines, decreased cell proliferation and ERK1/2 phosphorylation level were observed. Lastly, we also identified CYP3A43 co-expressed genes in LUAD from LinkedOmics database followed by GO and KEGG analyses. In conclusion, our results indicate the unprecedented role of CYP3A43 in the suppression of LUAD and provide new possibilities for targeted therapy of this life-threatening disease.

## 1. Introduction

Lung cancer is the leading cause of cancer death worldwide, and LUAD has been the most frequent histological subtype in recent years [1,2]. It has been reported that the development of LUAD is mainly caused by a combination of environmental factors and genetic alterations, in which the activation of proto-oncogenes and inactivation of onco-suppressor genes play a key role in the development of LUAD [3]. Over the past three decades, extensive efforts have been made in the treatment of LUAD concurrent with the emergence of precision medicine. Specifically, genome-guided molecularly targeted therapies have become hotspots in cancer research [4]. Molecularly targeted therapies prevent tumor growth by precisely inhibiting the signaling pathways that promote the malignant phenotype of cells at the gene-molecule level. Such therapies are highly targeted and have low toxic side effects [5,6,7]. Although most current treatments are deemed to be effective, the 5-year survival rate of LUAD patients remains unsatisfactory [8]. Hence, further studies are required to find out new and efficient biomarkers that can be used to screen high-risk patients and assess tumor progression in LUAD.

The CYP3A subfamily is the most vital group of CYP450s. This subfamily shows a very wide substrate specificity [9] and participates in the metabolism of more than half of the known drugs [10], endogenous steroids (e.g., testosterone, progesterone, and cortisol), and exogenous compounds [11]. CYP3A43, a member of the CYP3A subfamily, is less well-studied than the other three members (CYP3A4, CYP3A5, and CYP3A7) [12]. Recently, most studies on the association between CYP3A43 and tumors have focused on single nucleotide polymorphisms. Some studies have reported that the CYP3A43*3 genotype (P340A; rs680055) is correlated with an increased risk of prostate cancer [13,14]. A study on CYP3A43 polymorphisms in breast cancer showed a significant association between CYP3A43_74_delA (CYP3A43*2A, rs61469810) and tumor grade [15]. Moreover, CYP3A43 may be a double-facet molecule in tumors. One study reported that CYP3A43 may be a promising predictive marker for hepatocellular carcinoma, as low expression level of CYP3A43 in tumor tissues was associated with reduced median survival of patients [16]. Another study reported that CYP3A43 expression in ovarian cancer was higher than its expression in the normal ovary, but CYP3A43 was not identified as an independent prognostic marker of ovarian cancer [17]. The dual role of CYP3A43 indicates that it may participate in the development of cancer and that its function is tissue specific. However, the biological function of CYP3A43 in cancer is scarcely explored.

In our study, we examined the association between CYP3A43 expression and the clinicopathological features of LUAD patients. We report, for the first time to our knowledge, the unprecedented biological functions of CYP3A43 in suppressing the proliferation and migration of human lung cancer cells both in vitro and in vivo.

## 2. Results

### 2.1. Low CYP3A43 Expression Is Linked to Cancer Staging and Lymph Node Metastasis

**Figure 1:**
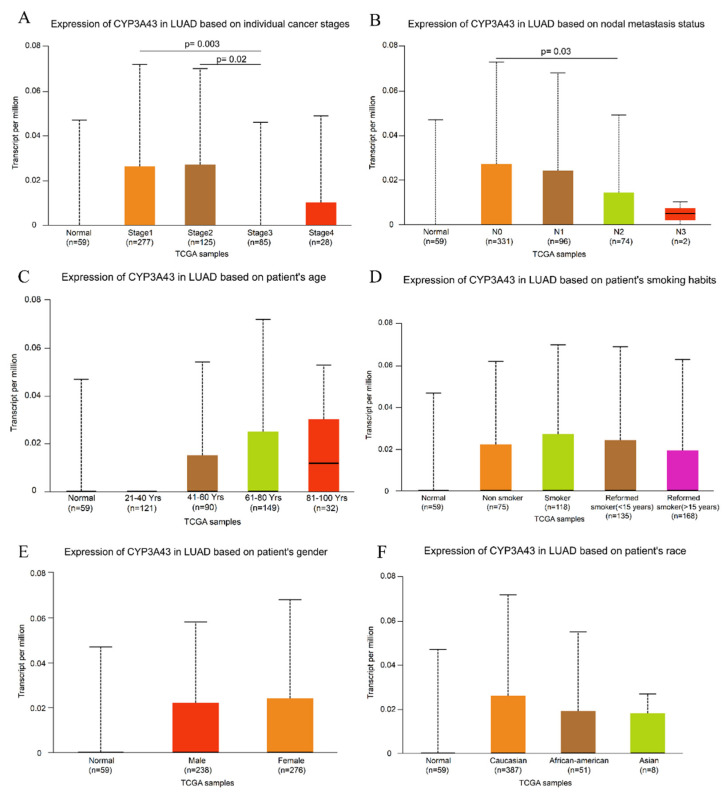
UALCAN portal results of the CYP3A43 expression level in different subgroups of LUAD. (**A**) Individual cancer stages; (**B**) nodal metastasis; (**C**) age; (**D**) smoking habits; (**E**) gender; (**F**) race.

To determine whether CYP3A43 is involved in the development of LUAD, we first studied the association between CYP3A43 expression and the clinicopathological characteristics of LUAD using the UALCAN database. We found a correlation between the expression level of CYP3A43 and the cancer staging and lymph node metastasis. Specifically, CYP3A43 levels in cancer samples from stage 3 patients were significantly lower than those in samples from stage 1 (*p* = 0.003) and stage 2 patients (*p* = 0.02), respectively (Figure 1A). Furthermore, decreased CYP3A43 expression was observed in tumor tissues with lymph node metastasis (*p* = 0.03) compared to tumor tissues without lymph node metastasis (Figure 1B). The CYP3A43 expression was not correlated with the gender, age, or smoking status of LUAD patients (Figure 1C–F). These results indicated that CYP3A43 expression was associated with the progression and malignancy of LUAD.

### 2.2. Stable CYP3A43 Knockdown Promotes Cell Proliferation In Vitro and Tumor Growth In Vivo

**Figure 2:**
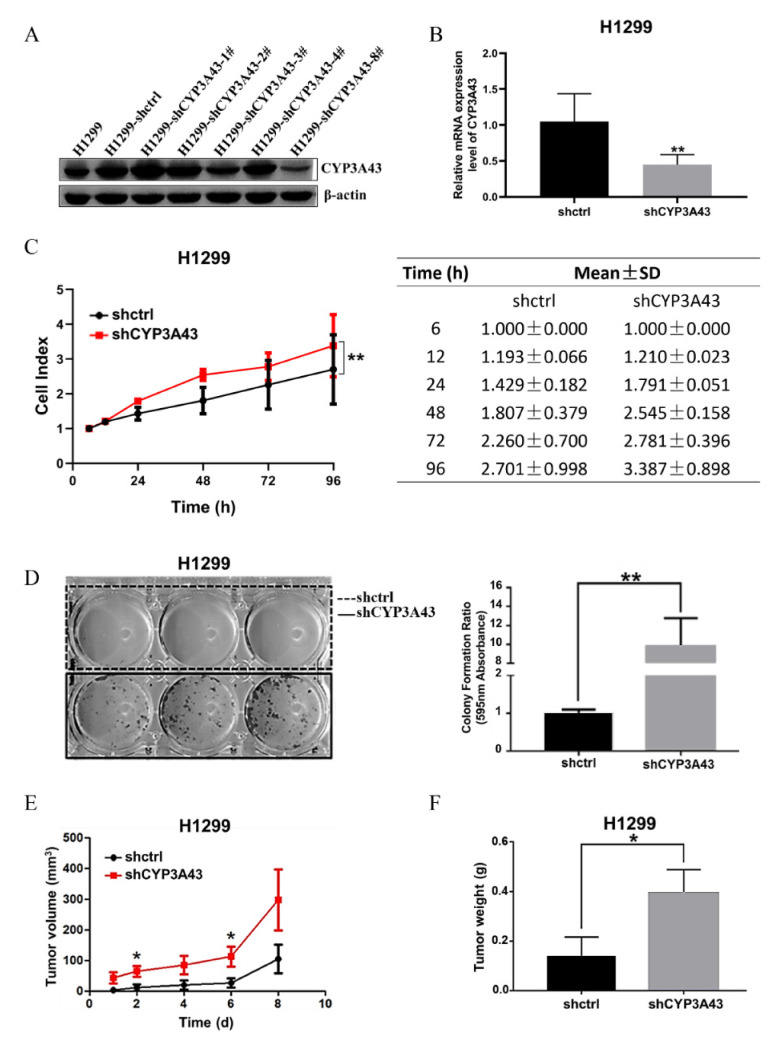
CYP3A43 knockdown facilitated the growth of H1299 cells both in vitro and in vivo. (**A**) The immunoblot results showed that CYP3A43 expression was suppressed by CYP3A43 shRNA, and H1299-shCYP3A43-8# had the strongest inhibitory effect. (**B**) The mRNA level of stable CYP3A43-knockdown H1299 cells and the shctrl cells was detected by RT-PCR analysis. (**C**) Real-time monitoring of CYP3A43 on the proliferation of H1299-shctrl and H1299-shCYP3A43 cells were confirmed by RTCA. The results are shown as the mean ± SD, *n* = 3. (**D**) Knockdown of CYP3A43 in H1299 cells significantly promoted colony formation ability compared to the shctrl cells. (**E**,**F**) Stable CYP3A43 knockdown in H1299 cells increased tumor growth (**E**) and tumor weight (**F**) (*n* = 10 mice for each injected group). *, *p* < 0.05; **, *p* < 0.01.

We first compared the mRNA and protein expression level of CYP3A43 in different human lung cancer cell lines to that in BEAS-2B cells (Supplementary Figure S1). To further investigate the function of CYP3A43 in the development of LUAD, we established CYP3A43-knockdown H1299 cell lines. The immunoblot results showed that CYP3A43 expression was dramatically reduced in the H1299-shCYP3A43-8# cells compared to the H1299-shctrl cells (Figure 2A). Therefore, the H1299-shCYP3A43-8# cells were selected to perform further experiments. The RT-PCR results indicated that CYP3A43 mRNA was significantly decreased in the stable CYP3A43-knockdown H1299 (vs. H1299-shctrl, *p* = 0.0048) (Figure 2B).

Next, we evaluated the effect of CYP3A43 expression on the H1299 cell viability by Real-Time Cell Analysis (RTCA). As shown in Figure 2C, CYP3A43 knockdown increased the proliferation of H1299 cells compared to control cells (*p* < 0.0027). To verify the long-term effect of CYP3A43 knockdown on cell survival, a colony formation assay was performed. As shown in Figure 2D, compared to the corresponding control cells, the relative colony formation rate of the stable CYP3A43-knockdown H1299 cells increased significantly. These data indicated that CYP3A43 knockdown promotes cell proliferation in vitro.

We further investigated the function of CYP3A43 on tumor growth in vivo using a xenograft mouse model. The stable H1299-shctrl and H1299-shCYP3A43 cells were injected subcutaneously into BALB/c nude mice. We found that CYP3A43 knockdown accelerated tumor growth and increased tumor weight (Figure 2E,F). These findings confirmed that CYP3A43 knockdown promotes the growth of H1299 cells both in vitro and in vivo.

### 2.3. CYP3A43 Overexpression Inhibits LUAD Cell Proliferation

**Figure 3:**
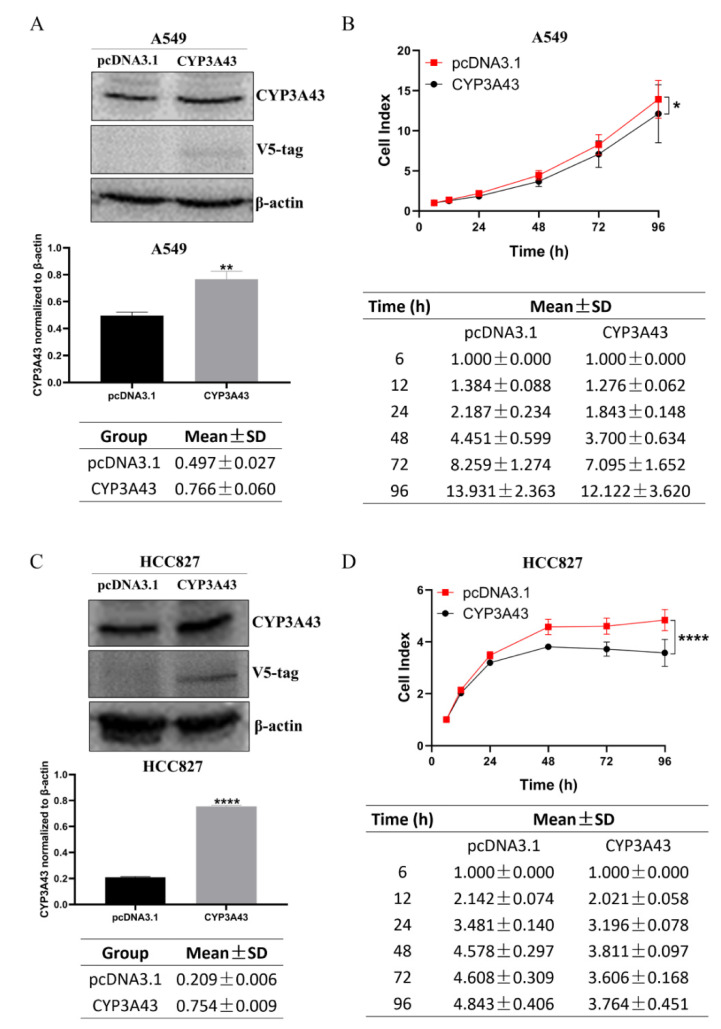
CYP3A43 inhibits the cell proliferation of LUAD cells. (**A**,**C**) The protein expression levels of CYP3A43 in A549 (**A**) and HCC827 (**C**) cells were detected after transfection with CYP3A43 plasmid for 24 h by immunoblot assay. (**B**,**D**) RTCA showed that the cell proliferation of A549 (**B**) and HCC827 (**D**) cells transfected with CYP3A43 plasmid was lower compared to the control cells. These results are shown as the mean ± SD, n = 3. *, *p* < 0.05; **, *p* < 0.01; ****, *p* < 0.0001.

We used the CYP3A43 plasmid to transiently overexpress CYP3A43 in A549 and HCC827 cells. The immunoblot results indicated that the protein expression of CYP3A43 in A549 and HCC827 cells transfected with CYP3A43 plasmid was higher than that in the corresponding vector control cells (Figure 3A,C). Moreover, the RTCA results showed that CYP3A43 overexpression significantly inhibited the cell proliferation ability of A549 and HCC827 cells (Figure 3B,D); however, the colony formation assay results indicated that overexpression of CYP3A43 did not affect the clonogenic ability of these cells (Supplementary Figure S2). Based on these results, we concluded that CYP3A43 negatively regulates the proliferation of LUAD cells.

### 2.4. CYP3A43 Knockdown Promotes H1299 Cell Migration

**Figure 4:**
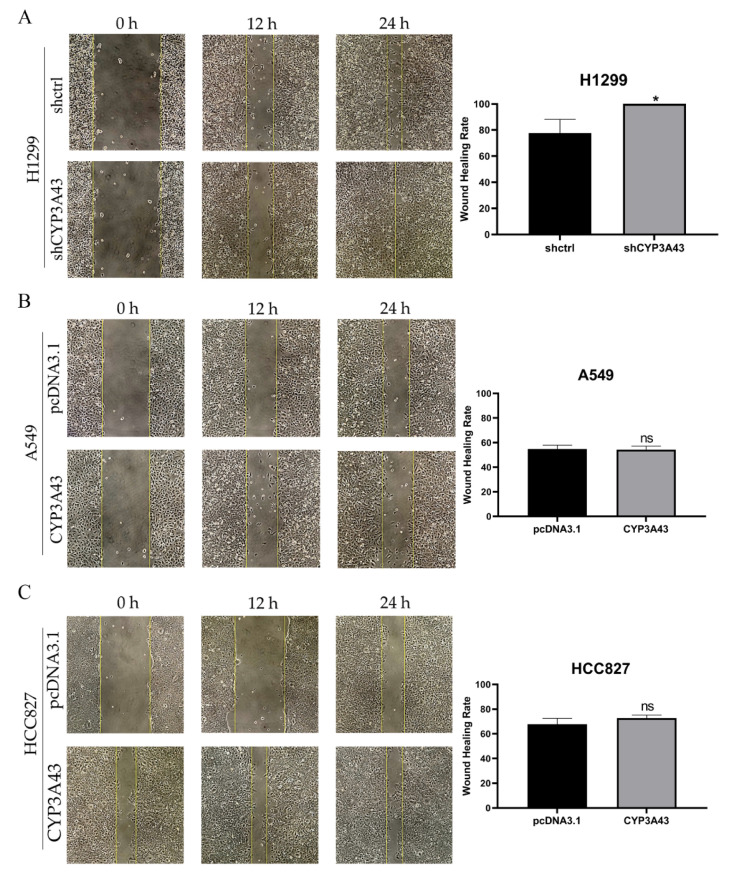
CYP3A43 knockdown promotes the migration ability of H1299 cells. (**A**) The cell migration ability of H1299-shCYP3A43 cells was increased compared to the H1299-shctrl cells. (**B**,**C**) CYP3A43 overexpression did not affect the migration ability of A549 (**B**) and HCC827 (**C**) cells. The A549 and HCC827 cells were transfected with CYP3A43 plasmids or empty vector for 24 h, and then a wound healing assay was performed. The results are shown as the mean ± SD, *n* = 3. *, *p* < 0.05; ns, *p* > 0.05.

One of the main reasons for the high rate of recurrence and mortality from LUAD is the high metastatic potential of these tumor cells [18]. Based on the above which we found that CYP3A43 underexpression was significantly associated with lymph node metastasis in LUAD patients, and stable CYP3A43 knockdown could enhance the proliferation of H1299 cells in vitro and in vivo; next, we determined the role of CYP3A43 in the migration ability of LUAD cells. The wound healing assay results showed that CYP3A43 knockdown increased the migration ability of H1299 cells (Figure 4A), whereas CYP3A43 overexpression had no effect on the migration ability of A549 or HCC827 cells (Figure 4B,C).

### 2.5. Gene Set Enrichment Analysis of CYP3A43 Co-Expressed Genes in LUAD

**Figure 5:**
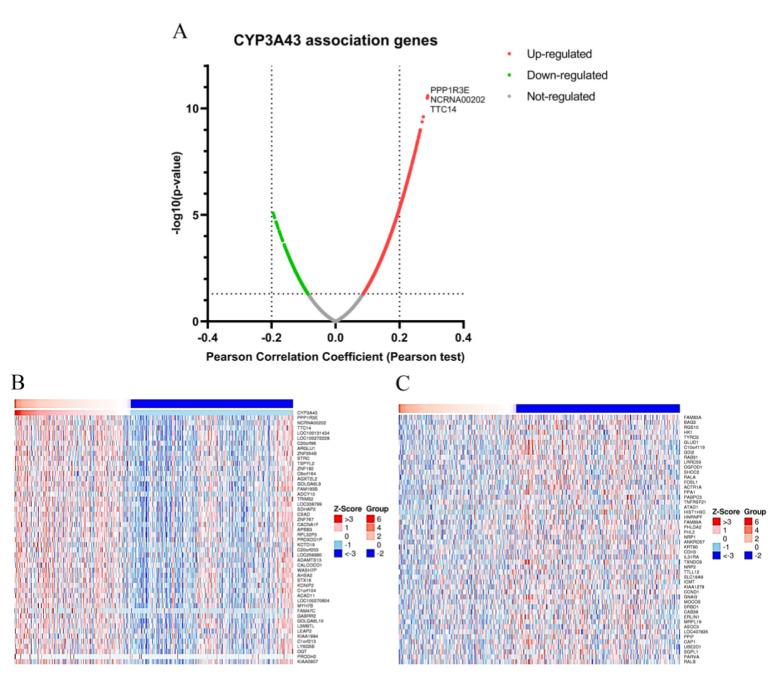
CYP3A43 co-expressed genes in LUAD. (**A**) Volcano plot showing the CYP3A43 co-expressed genes. (**B**,**C**) Heatmaps showing the top 50 significant genes positively (**B**) and negatively (**C**) associated with CYP3A43 in LUAD.

To further investigate the function of CYP3A43 in LUAD, the genes co-expressed with CYP3A43 were identified using the LinkedOmics database. Figure 5A shows that the expression of protein phosphatase 1 regulatory subunit 3E (PPP1R3E), family with sequence similarity 238 member C (NCRNA00202), and tetratricopeptide repeat protein 14 (TTC14), has the strongest positive correlation with CYP3A43 expression in LUAD (PPP1R3E: Pearson’s r = 0.3; NCRNA00202: Pearson’s r = 0.28; TTC14: Pearson’s r = 0.28). We identified 893 differential genes with FDR < 0.01 and adjusted *p* < 0.05 as screening conditions (Supplementary Tables S1 and S2). Overall, 869 genes were positively correlated with CYP3A43 expression, whereas 24 genes were negatively correlated with CYP3A43 expression. Heatmaps of the top 50 significant genes both positively and negatively correlated with CYP3A43 expression are shown in Figure 5B,C.

**Figure 6:**
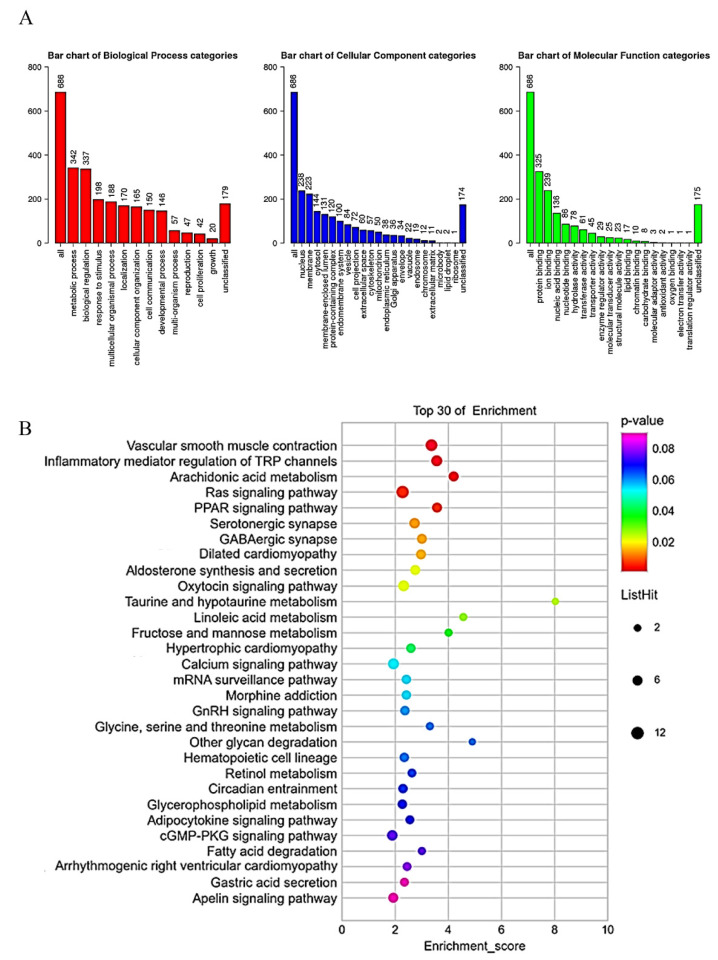
GO and KEGG enrichment analysis of CYP3A43 co-expressed genes. (**A**) Gene ontology analysis of the genes co-expressed with CYP3A43 in LUAD. (**B**) Bubble plot for the KEGG analysis of the genes co-expressed with CYP3A43 in LUAD. Bubble color represents *p* value. Bubble diameter represents gene count.

We performed GO and KEGG analyses to further explore the possible gene functions and signaling pathways in which CYP3A43 might be involved. The GO analysis results suggested that the CYP3A43 co-expressed genes were mainly located in the nucleus and membrane; these genes were involved in metabolic processes, biological regulation, and responding to stimuli through molecular functions such as protein binding, ion binding, and nucleic acid binding (Figure 6A). The KEGG pathway analysis showed that the CYP3A43 co-expressed genes were most likely participated in vascular smooth muscle contraction, regulation of transient receptor potential (TRP) channels by inflammatory mediators, and the Ras signaling pathway. Interestingly, the Ras signaling pathway included the highest number of the identified co-expressed genes (Figure 6B).

### 2.6. Involvement of CYP3A43 in ERK1/2 Signaling

**Figure 7:**
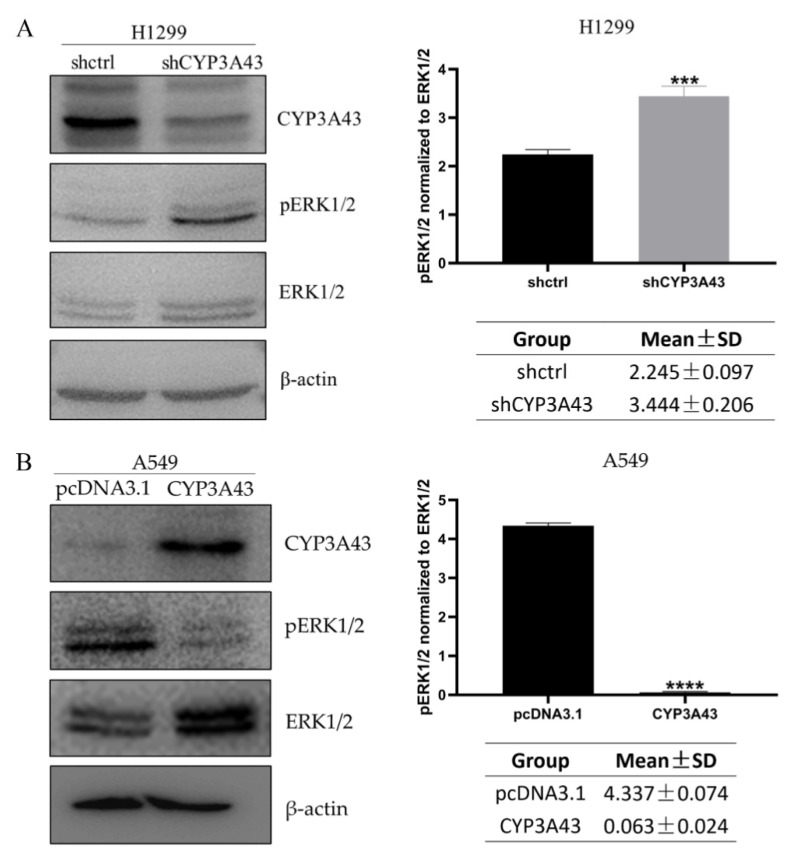
The expression of CYP3A43 affects ERK1/2 phosphorylation. (**A**) Increased ERK1/2 phosphorylation levels were detected in H1299-shCYP3A43 cells compared to the control group. (**B**) Decreased ERK1/2 phosphorylation levels were detected in A549-CYP3A43 cells compared to the control group. These results are shown as mean ± SD, *n* = 3. ***, *p* < 0.001; ****, *p* < 0.0001.

Previous studies have shown that the ERK1/2 is a critical downstream target of Ras, and the Ras-MEK1/2–ERK1/2 pathway is an important signaling pathway that regulates a variety of biological functions, including proliferation, migration, invasion, etc. [19]. Therefore, we conducted an experiment to verify if ERK1/2 phosphorylation changes under the conditions of CYP3A43 knockdown and overexpression. Our results showed that the CYP3A43 knockdown conditions supported an increase in the ERK1/2 phosphorylation level of H1299-shCYP3A43 cells compared with the shctrl cells (Figure 7A). However, the overexpression of CYP3A43 in A549 cells appeared to inhibit ERK1/2 phosphorylation (Figure 7B). Activated ERK1/2 led to phosphorylation of Elk1, c-Jun, c-Myc, and SP1 transcription factors, which regulate genes related to the cell cycle and cell proliferation [20]. Our findings indicate that the CYP3A43-induced proliferation and migration of LUAD cells might be dependent on ERK1/2 signaling.

**Figure 8:**
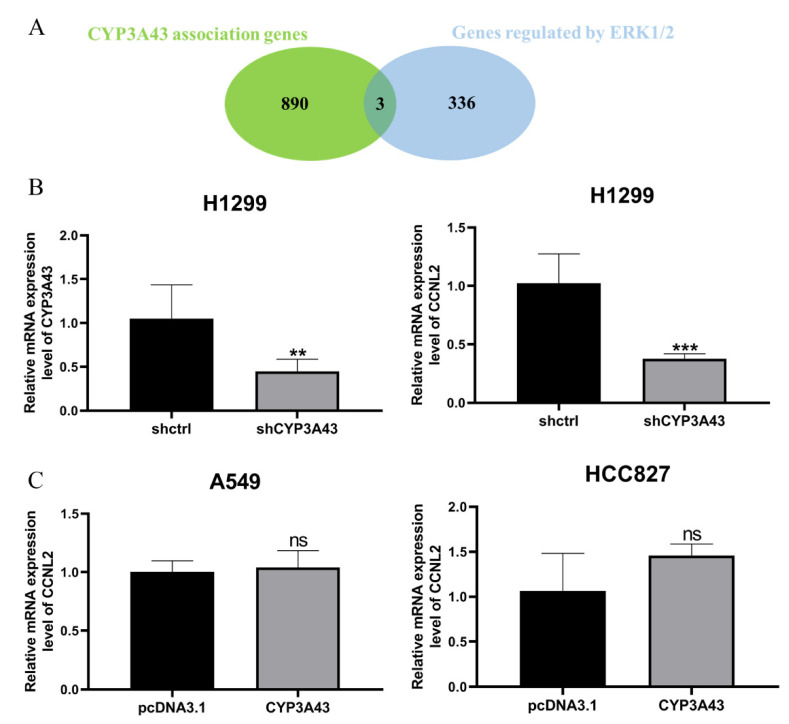
CCNL2 expression was decreased in the stable CYP3A43-knockdown H1299 cells. (**A**) A Venn diagram showing the number of overlapping genes between the two gene sets (CYP3A43 co-expressed genes and genes that may be regulated by the ERK1/2 downstream transcription factors). (**B**) Decreased CCNL2 mRNA levels were detected in the H1299 cells with stable knockdown of CYP3A43 expression. (**C**) CYP3A43 overexpression in the A549 and HCC827 cells had no effect on CCNL2 mRNA expression. **, *p* < 0.01; ***, *p* < 0.001; ns, *p* > 0.05.

In addition, three overlapping genes (integrator complex subunit 3 (INTS3), cyclin L1 (CCNL1), and cyclin L2 (CCNL2)) were screened with overlapping gene identification using the co-expressed genes of CYP3A43 and some genes that may be regulated by the downstream transcription factors of ERK1/2 (Figure 8A). The RT–PCR experiments found that CCNL2 mRNA expression was decreased in the H1299-shCYP3A43 cells compared to the H1299-shctrl cells (Figure 8B), whereas the mRNA expression level of the other two genes was not influenced by the CYP3A43 expression (Supplementary Figure S3). However, there was no difference in the mRNA expression level of CCNL2 between the CYP3A43-transfected group and the vector transfection group (Figure 8C). These results suggested that CYP3A43 knockdown may affect CCNL2 expression through the ERK 1/2 pathway.

## 3. Discussion

Previous reports have shown that CYP450s are generally considered to have an important function in tumor progression through the activation of carcinogens and the metabolism of anticancer drugs [21]. Ho et al. showed that CYP2E1 expression is down-regulated in liver cancer, and its down-regulation is related to tumor aggressiveness and poor prognosis of patients. Their results suggest that the differential expression of CYP2E1 may play an important role in the occurrence of liver cancer [22]. Other studies have reported that CYP2A13 expression is down-regulated in LUAD, indicating that CYP2A13 may participate in the occurrence and development of LUAD [23]. Therefore, the activity and expression level of CYP450s play a crucial role in cancer risk, tumorigenesis, and chemoprophylaxis. In our study, we focused on the role of CYP3A43 in the development of LUAD and found that CYP3A43 is closely related to the development of LUAD.

In our analysis, CYP3A43 expression was negatively associated with the clinical stage and lymph node metastasis of LUAD patients. As increased cellular proliferation and migration are the hallmarks of cancer cells [24], the observation that CYP3A43 knockdown promotes proliferation and migration of H1299 cells provides evidence to address how CYP3A43 negatively affects LUAD patients’ cancer stage and lymph node metastasis. Our in vivo experimental results demonstrated that CYP3A43 knockdown also significantly accelerates tumor growth. These findings further verified that CYP3A43 knockdown could promote the tumorigenesis and growth of LUAD. Based on the observation that CYP3A43 knockdown promotes the proliferation and migration of H1299 cells, we suspect that CYP3A43 overexpression could block and inhibit cancer. Although overexpressed CYP3A43 in A549 and HCC827 cells could inhibit cell proliferation, its overexpression had no effect on the clonogenic or migration ability of these cells, further investigation is underway to clarify these observations.

We investigated the potential targets of CYP3A43 to better understand the molecular mechanism by which CYP3A43 expression regulates carcinogenesis and progression of LUAD. Our results showed that PPP1R3E, NCRNA00202, and TTC14 expression had a strong positive correlation with the expression of CYP3A43. However, the role of these genes in LUAD has not been studied. PPP1R3E is a central regulator of important cellular events such as cell cycle progression, apoptosis, and DNA damage response [25,26]; while NCRNA00202 and TTC14 have not been well-studied to date.

The proliferation and metastasis of LUAD are regulated by various signaling pathways [27,28]. Our GO and KEGG analyses demonstrated that the genes co-expressed with CYP3A43 were mainly involved in the Ras signaling pathways. The Ras signaling pathways are significant kinase module pathways, which are involved in many cellular processes such as cell proliferation, differentiation, migration, and apoptosis [29,30]. The RAF–MEK–ERK pathway, the most classic of the RAS signaling pathways, could regulate the proliferation, migration, and apoptosis of tumor cells, which are crucial to the occurrence and progression of cancer [31]. ERK1 and ERK2, serine and threonine kinases, are two important members of the MAPK/ERK pathway that play roles in regulating tumor cell proliferation, migration, and apoptosis [31]. ERK1/2 is located in the cytoplasm of unstimulated cells. Once activated, ERK1/2 is transferred to the nucleus where it regulates the activity of various transcription factors (such as Elk1, c-Fos, c-Jun, and c-Myc) through phosphorylation, thereby contributing to tumorigenesis or cancer growth [32]. A previous study showed that ERK1/2 is activated in non-small cell lung cancer (NSCLC) and that ERK1/2 activation is positively correlated with cancer stage and lymph node metastasis [33]. It has also been reported that the proliferation and metastasis of NSCLC cells (NSCLCs) could be suppressed via ERK1/2 inhibition [34,35,36]. Thus, we suspected that CYP3A43 plays a role in the ERK1/2 pathway. Indeed, our results indicated that CYP3A43 knockdown promotes cell proliferation and migration by targeting the MAPK signaling pathway as the phosphorylation of ERK1/2 was markedly elevated in the CYP3A43-knockdown H1299 cells. Accordingly, we also discovered that CYP3A43 overexpression inhibits the activation of ERK1/2, which suggested that CYP3A43 overexpression suppresses NSCLC cell growth by inhibiting the ERK1/2 activation. In other words, our study provides possible molecular evidence that CYP3A43 underexpression promotes the proliferation and migration of LUAD.

We used online database to further study the regulatory network of CYP3A43 in the ERK1/2 pathway. Three overlapping genes, INTS3, CCNL1 and CCNL2, were obtained by searching for the intersections between CYP3A43 coexpressed genes and some genes that may be regulated by downstream transcription factors of ERK1/2. Among these three genes, CCNL2 had a significantly lower mRNA expression in the CYP3A43-knockdown H1299 cells. CCNL2 was reported to promote apoptosis of human hepatoma cells in vitro [37]. CCNL2 overexpression is also known to induce apoptosis and cell cycle arrest of human lung cancer cells [38]. Therefore, we hypothesized that CYP3A43 knockdown may decrease the mRNA expression of CCNL2 by activating the ERK1/2 pathway, thus promoting cell proliferation. However, further experiments are needed to confirm this hypothesis.

There are some limitations in this study. First, it remains unclear whether ERK1/2 is directly or indirectly activated by CYP3A43 knockdown. Second, we found that CYP3A43 knockdown can promote the proliferation and migration of LUAD cells and tumorigenesis in vivo, but CYP3A43 overexpression does not inhibit the clonal formation and migration of LUAD cells. It is also unclear whether the enzymatic activity of CYP3A43 is involved in all these processes. Nevertheless, our findings strongly demonstrate that CYP3A43 regulates the proliferation and migration of LUAD cells through the modulation of ERK1/2 signaling.

## 4. Materials and Methods

### 4.1. UALCAN Database

The UALCAN (http://ualcan.path.uab.edu/cgi-bin/ualcan-res.pl) is a very comprehensive platform containing clinical data on multiple cancer types from the TCGA dataset, allowing analysis of gene expression in correlation with clinical features of tumors [39,40]. In this study, we used the UALCAN database to investigate the correlation between CYP3A43 mRNA expression and clinicopathological features of LUAD on 15 April 2021. We accessed the UALCAN website and then extracted the data using the following steps: (1) clicked on “TCGA analysis”, (2) entered “CYP3A43” in the “Enter gene symbol(s)” section and “LUAD” in the “TCGA dataset” section, (3) selected “Expression” in the pop-up page, and (4) clicked “Explore”. The differences in CYP3A43 mRNA expression between different subgroups were analyzed by selecting cancer stage, lymph node metastasis, patient race, age, gender, and smoking habit.

### 4.2. Cell Culture and Reagents

The human LUAD cell lines H1299, A549, and HCC827 were obtained from the ATCC Cell Bank of the Chinese Academy of Sciences. The H1299 and HCC827 cell lines were cultured in RPMI-1640 medium with 10% fetal bovine serum and 1% Penicillin-Streptomycin at 37 °C in a 5% CO_2_ incubator. The A549 cell line was cultured in F12K medium supplemented with 10% fetal bovine serum and 1% Penicillin-Streptomycin at 37 °C in a 5% CO_2_ incubator.

### 4.3. Plasmids, shRNA, and Transfection

The CYP3A43 expression plasmid pcDNA3.1-V5-His A-CYP3A43 was obtained from IGE BIOTECHNOLOGY LTD. The vector plasmid pcDNA3.1/V5-His A was purchased from Invitrogen. The short-hairpin RNA targeting human CYP3A43 (Forward oligo

5′-CCGGGCCTGGTACTCCTCTATATTTCTCGAGAAATATAGAGGAGTACCAGGCTTTTTG-3′

Reverse oligo

5′-AATTCAAAAAGCCTGGTACTCCTCTATATTTCTCGAGAAATATAGAGGAGTACCAGGC-3′) was synthesized by IGE BIOTECHNOLOGY LTD, and then incorporated into a pLKO.1 TRC cloning vector (Cat. #10878, Addgene) following the protocol provided by the manufacturer.

The transfections were performed with PEI reagent according to the manufacturer’s instructions. The trypsinized single cells in a medium with 10% FBS were transfected with plasmids or shRNA and cultured for 24 h before the cells were used for subsequent analysis.

### 4.4. Establishment of the CYP3A43-knockdown H1299 Cell Lines

The H1299 cells were transfected with pLKO.1-shctrl and pLKO.1-shCYP3A43 in 10 cm dishes. Puromycin was used to screen for vector-containing cells. An immunoblot analysis was performed to examine the CYP3A43 expression level. The CYP3A43-knockdown cells were called H1299-shCYP3A43.

### 4.5. Cell Index Assessment

The cell proliferation was measured using the xCELLigence Real-Time Cell Analysis (RTCA) instrument (ACEA Biosciences). Cells were seeded in 16-well E-plates, and the cell index was recorded every 15 min and continued for 96 h until the cells reached the plateau stage. The cell index was normalized (normalized cell index).

### 4.6. Colony Formation Assay

The cells were seeded into 6-well plates and cultured at 37 °C in a humidified incubator with 5% CO_2_ for 14 days. The medium was changed every three days, and the cell colonies were visible (>50 cells/colony) approximately 14 days later. The colonies were washed with 1×PBS for 3 times and fixed with methanol for 10 min. After 10 min, 0.5% crystal violet in 25% methanol (1×PBS) was added to each well and incubated for about 10–30 s. The crystal violet solution was discarded, and the colonies were washed with water 3 times and allowed to dry. The stained cell colonies were photographed and then eluted with acetic acid (10%, v/v). The absorbance of the eluent was measured at 595 nm.

### 4.7. Wound Healing Assay

The cells were seeded in 6-well plates and cultured to full confluence. Then, the cells were scratched using a 200-µL pipette tip and washed 3 times using DPBS. Subsequently, the cells were incubated in a medium containing 2% FBS. The wound healing process was captured using a phase-contrast microscope at the indicated time after injury. The area of wound healing was quantified using Image J software.

### 4.8. In Vivo Xenograft Assay

BALB/c Nude mice were purchased from Beijing Vital River Laboratory Animal Technology (Beijing, China). The mice were housed with a 12 h light/dark cycle and allowed free access to normal food and water. All animal studies were performed following the guidelines approved by the Shantou University Medical College Institutional Animal Care and Use Committee. The ethical code is SUMC2019-321.

To evaluate the role of CYP3A43 in the proliferation of H1299 cells in vivo, ten BALB/c Nude mice aged 6-week-old were randomly divided into two groups (n = 5 per group). All mice in each group received either H1299-shctrl or H1299-shCYP3A43 cells (4 × 10^6^ cells in 200 μL of DPBS) via subcutaneous injection into the right limb. The tumor size was measured every two days using digital calipers. Measurements of the tumor were taken manually by collecting the longest dimension (length) and the longest perpendicular dimension (width) of the tumor. The tumor volume was calculated with the formula V = (L × W^2^)/2 (V = volume, L = length and W = width). The tumor weight was analyzed using the Mann–Whitney test.

### 4.9. LinkedOmics Database

The LinkedOmics database (http://www.linkedomics.org/login.php) is a comprehensive online database based on the TCGA dataset [41]. This database was used to investigate the potential function of CYP3A43 in LUAD by identifying the co-expressed genes of CYP3A43 in TCGA-LUAD on 5 November 2021. We accessed the data used in this investigation with the following steps: (1) logged into the LinkedOmics website and registered, (2) selected the tumor type as “TCGA-LUAD”, (3) selected the data type as “RNA-seq”, (4) selected the gene to be analyzed as “CYP3A43“, (5) chose the target dataset as “RNA-seq” and the statistical method as “Pearson correlation test”, and (6) clicked the circle to browse and downloaded the resulting graph after the loading result showed “complete”.

### 4.10. GO and KEGG Analysis

The GO analysis was performed using the WebGestal (http://www.webgestalt.org/option.php) [42] online tool. We completed the GO analysis using the following steps: (1) entered the WebGestalt website and selected “Homo sapiens” for the Organism of Interest, (2) selected “Gene Set Enrichment Analysis (GESA)” for the Method of Interest, (3) selected “Functional Database” and “pathway, KEGG” for the Functional Database, (4) selected “Gene symbol” for the Gene ID Type, and (5) uploaded the list of CYP3A43 co-expressed genes and clicked “submit”. The results of the GO analysis are displayed in bar graphs.

For the KEGG analysis, we used the OECloud tools (https://cloud.oebiotech.cn). We completed the KEGG analysis using the following steps: (1) entered the website, (2) selected the GO/KEGG bubble chart, and (3) uploaded the list of CYP3A43 co-expressed genes to get the analysis results. The results of the KEGG analysis are presented as bubble charts.

### 4.11. RNA Extraction, Reverse Transcription, and RT-qPCR

The total RNA from cultured cells was extracted using the TRIzol reagent (Invitrogen). Then, a reverse transcription reaction was performed following the instructions from the Promega GoScript Reverse Transcription System (Promega). The target genes were amplified with specific primers and the GoTaq qPCR Master Mix (Promega). Relative gene expression was analyzed according to the comparative Ct method (2^−∆∆CT^ method), and β-actin was used as an internal control for each target gene.

All primers were obtained from IGE BIOTECHNOLOGY LTD (Guangzhou, China), with the following sequences: CYP3A43 (forward primer, 5′-TTTTACCCAATAAGGCACCTGT-3′; reverse primer, 5′-TTCTTGCAGACTCTCGTAACTC-3′), CCNL1 (forward primer, 5′-AGCCTCCAAGCCATCATCAC-3′; reverse primer, 5′-TCTTGCTGTCTTTTCTTACACCA-3′), CCNL2 (forward primer, 5′-GTACTCCGGGGTGCTCATC-3′; reverse primer, 5′-GAGGTCGGTCTCTGTGTCG-3′), INTS3 (forward primer, 5′-GGGCAATGCTGAGAGAGAAG-3′; reverse primer, 5′-TGCCTCTGCATTGTCATAGC-3′), and β-actin (forward primer, 5′-GAGCTACGAGCTGCCTGACG-3′; reverse primer, 5′-CTCCATGCCCAGGAAGGAAGG-3′).

### 4.12. Protein Extraction and Immunoblot Analysis

The cells were washed with ice-cold PBS and then scraped from the surface of the culturing dishes and collected by centrifugation. Total protein lysates were extracted using the RIPA lysis buffer containing protease inhibitors and phosphatase inhibitors. Then, a Bradford assay was used to estimate protein concentrations. The protein samples were loaded onto 10% SDS-PAGE gels and transferred to a PVDF membrane. The membranes were blocked with 5% nonfat milk in Tris-Buffered Saline (TBS) containing 0.05% Tween 20 for 1.5 h and then incubated overnight with the primary antibodies at 4 °C. Antibodies against CYP3A43 (GTX117120, 1:2000 dilution, GeneTex, Irvine, CA, USA), the mouse monoclonal antibodies against human β-actin (1:20,000, A5441, Sigma Aldrich, shanghai, China) were used in our study. The following antibodies were obtained from Cell Signaling Technology (CST) (Danvers, MA, USA): rabbit monoclonal antibodies against human p-ERK T202/Y204 (#4370, 1:1000 dilution) and ERK1/2 (#9102, 1:1000 dilution). On the next day, after washing three times with 1×TBST, the membranes were incubated for 1.5 h with HRP-conjugated secondary antibodies at room temperature. The protein signals were visualized using Enhanced Chemiluminescence Western Blotting Detection Reagents (GE Healthcare) under a Chemiluminescent Imaging System (Tanon 5200).

### 4.13. Statistical Analysis

The two-way ANOVA and Student’s *t*-test were used in the statistical analyses. Graphpad Prism 8 was used for all data analysis and graph generation.

## 5. Conclusions

In summary, the current study showed that CYP3A43 expression was negatively correlated with the clinical stage and lymph node metastasis of LUAD patients. CYP3A43 knockdown increased the proliferation, colony formation, and migration of H1299 cells. CYP3A43 knockdown also significantly promoted tumor growth in a xenograft mouse model. Conversely, the overexpression of CYP3A43 inhibited the proliferation of A549 and HCC827 cells. Mechanistically, we believe CYP3A43 knockdown somehow leads to the activation of the ERK1/2 pathway. However, to absolutely clarify the function of CYP3A43 in tumorigenesis, future studies should be conducted to connect the missing links. Overall, our results suggest that CYP3A43 might serve as a potential therapeutic target for blocking LUAD development.

## Data Availability

All the data presented in this study are available upon request from the corresponding author.

## Supplementary Materials

The following supporting information can be downloaded at: https://www.mdpi.com/article/10.3390/ijms24010113/s1.
