# Supplementary material for: Effects of CYP3A43 Expression on Cell Proliferation and Migration of Lung Adenocarcinoma and Its Clinical Significance
Source: Int J Mol Sci. 2022 Dec 21;24(1):113. doi: 10.3390/ijms24010113 (PMC9820144; doi:10.3390/ijms24010113)
Supplement: Supplementary file 1 [file ijms-24-00113-s001.zip › ijms-1974926-supplementary.pdf]

Supplementary Materials

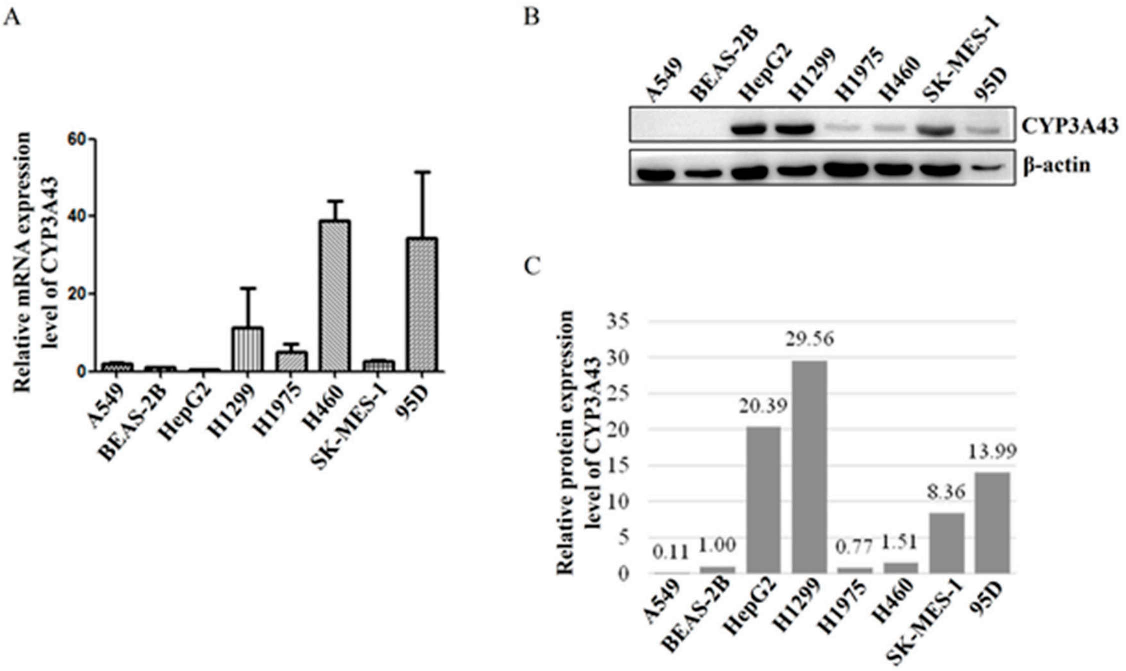

Supplementary Figure S1. The mRNA and protein expression levels of CYP3A43 in experimental cell lines (**A**) The relative mRNA expression levels of CYP3A43 in BEAS-2B and seven cancer cell lines by RT-qPCR. (**B,C**) Immunoblot results of CYP3A43 in BEAS-2B and seven cancer cell lines. The relative protein expression levels of CYP3A43 are shown after normalization with β-actin (setting 1 for BEAS-2B).

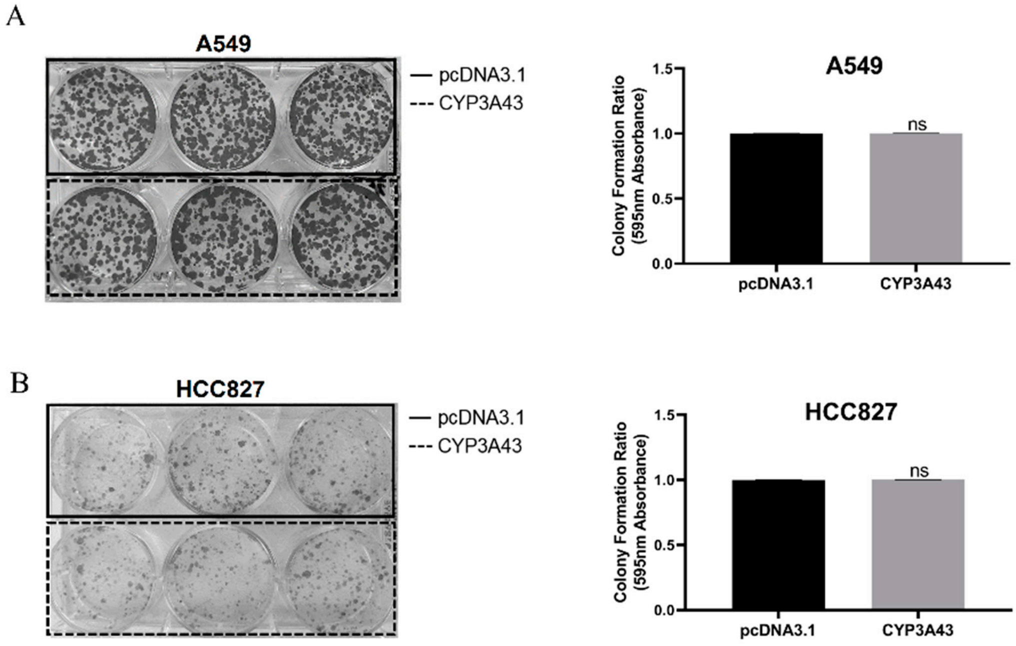

Supplementary Figure S2. CYP3A43 overexpression did not affect the colony formation ability of A549 and HCC827 cells. The A549 (**A**) and HCC827 (**B**) cells were transfected with plasmid encoding CYP3A43

or empty vector pcDNA3.1 for 24 h, and a colony formation assay was performed. The colony formation ratios were determined and presented on the right. ns, $p > 0.05$.

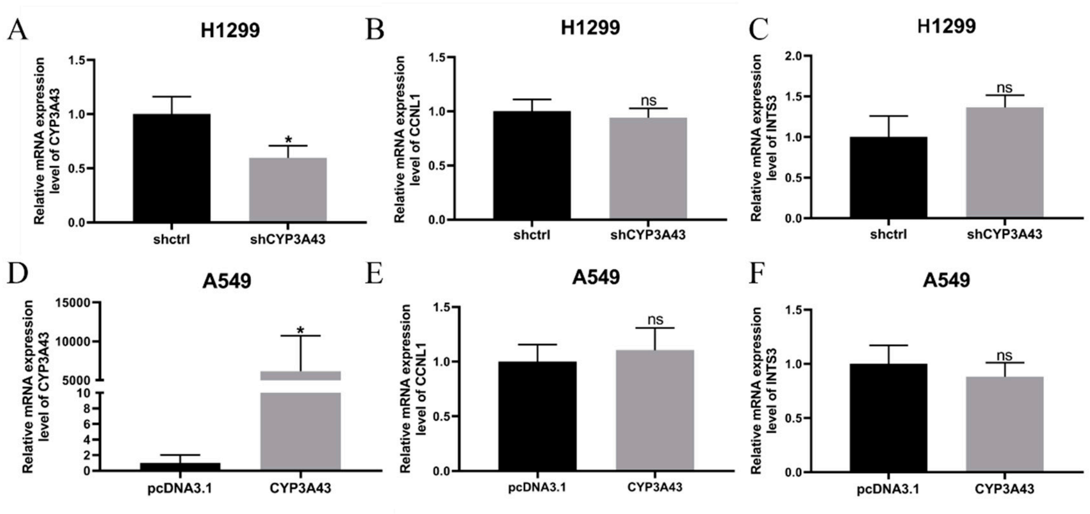

Supplementary Figure S3. CYP3A43 did not regulate the expression of CCNL1 and INTS3. (**A-C**) Real-time PCR showed that CCNL1 and INTS3 mRNA levels were not altered in CYP3A43 knockdown H1299 cells. (**D-F**) Real-time PCR showed that CCNL1 and INTS3 mRNA levels were not altered in CYP3A43 overexpressed A549 cells. *, $p < 0.05$; ns, $p > 0.05$.

Supplementary Table S1. The correlation coefficients between CYP3A43 and its co-up-regulated gene.

Gene	Pearson CC	Gene	Pearson CC	Gene	Pearson CC	Gene	Pearson CC	Gene	Pearson CC
**PPP1R3E**	0.30	C16orf3	0.21	ZNF711	0.19	EFHC1	0.18	LTB4R2	0.16
**NCRNA00202**	0.29	LENG8	0.21	SPTBN4	0.19	FER1L4	0.18	NISCH	0.16
**TTC14**	0.29	TRIM66	0.21	RBM39	0.19	C14orf181	0.18	SGSM2	0.16
**LOC100131434**	0.29	SPIN3	0.21	FLJ40330	0.19	C9orf96	0.18	PRKCG	0.16
**LOC100272228**	0.27	GRIPAP1	0.21	PDXDC2	0.19	WDR90	0.18	LOC55908	0.16
**C20orf96**	0.27	KIAA1875	0.21	ACACB	0.19	LOC400891	0.17	NHLRC3	0.16
**ARGLU1**	0.27	DND1	0.21	CES8	0.19	ZNF141	0.17	CLCN6	0.16
**ZNF354B**	0.27	ZNF589	0.21	FAM153A	0.19	RABL2A	0.17	MRI1	0.16
**STRC**	0.26	ANKRD10	0.21	CACNB4	0.19	ZNF224	0.17	FAM153C	0.16
**TSPYL2**	0.26	SPEF2	0.21	TMEM120B	0.19	ATP2B2	0.17	ANKS1B	0.16
**ZNF182**	0.26	C1orf152	0.21	JRK	0.19	SPDYE6	0.17	MGC21881	0.16
**C6orf164**	0.26	C6orf134	0.21	SYNGAP1	0.19	SLC25A36	0.17	GLYCTK	0.16
**AGXT2L2**	0.26	GOLGA6B	0.21	SLC2A4	0.19	SEC16B	0.17	CT45A2	0.16
**GOLGA6L9**	0.26	UBXN11	0.21	CBFA2T2	0.19	ZFC3H1	0.17	H2AFB1	0.16

**FAM193B**	0.26	C3orf35	0.21	FLCN	0.19	DUSP28	0.17	PPY2	0.16
**ADCY10**	0.26	RMND5B	0.21	ZNF540	0.19	LOC100216545	0.17	C14orf93	0.16
**TRIM52**	0.26	MYBPC3	0.21	GOLGA6L5	0.19	OGFOD2	0.17	ZXDC	0.16
**LOC338799**	0.26	PI4KAP1	0.21	SCN11A	0.19	GPCPD1	0.17	SRRM5	0.16
**SDHAP2**	0.26	HCG27	0.21	KIAA0895L	0.19	ZC3H12B	0.17	MDH1B	0.16
**CSAD**	0.26	MSTO2P	0.21	ABHD1	0.19	LOC284440	0.17	ZNF519	0.16
**ZNF767**	0.26	CYP2E1	0.21	TMEM116	0.19	LCNL1	0.17	MAN2C1	0.16
**CACNA1F**	0.26	C2orf57	0.21	TSSK3	0.19	HSD17B14	0.17	SENP7	0.16
**APBB3**	0.26	HTR3D	0.21	LOC150776	0.19	S100PBP	0.17	SNORD1C	0.16
**RPL32P3**	0.26	LOC100132287	0.21	FCHSD1	0.19	IP6K2	0.17	SLC26A1	0.16
**PRDXDD1P**	0.25	PHF1	0.21	KLKB1	0.19	ZNF514	0.17	C17orf65	0.16
**KCTD19**	0.25	FLJ45340	0.21	TUBGCP6	0.19	USP30	0.17	COL27A1	0.16
**C20orf203**	0.25	RFPL3S	0.21	ARIH2	0.19	WDR27	0.17	CCDC130	0.16
**LOC256880**	0.25	CLCNKA	0.21	RGL3	0.19	PDZK1P1	0.17	SLIT1	0.16
**ADAMTS13**	0.25	CDK5RAP3	0.21	SERPINA10	0.19	LOC146880	0.17	NXF1	0.16
**CALCOCO1**	0.25	SLC25A27	0.21	PRSS37	0.19	LEKR1	0.17	LRGUK	0.16
**WASH7P**	0.25	METTL3	0.21	CCDC159	0.19	TMEM175	0.17	GDAP1L1	0.16

**AHSA2**	0.25	PRAMEF6	0.21	DUSP15	0.19	SFI1	0.17	SLC22A11	0.16
**STX16**	0.25	ACBD4	0.21	LOC339047	0.19	NEURL3	0.17	PNMA5	0.16
**KCNIP2**	0.25	UBQLNL	0.21	JMJD7-PLA2G4B	0.19	NPEPL1	0.17	LOC91450	0.16
**C1orf104**	0.25	UPK1A	0.21	KLRA1	0.19	REM2	0.17	ADAT2	0.16
**ACAD11**	0.25	C3orf32	0.21	MALAT1	0.19	DFNB59	0.17	SATB1	0.16
**LOC100270804**	0.25	THSD1P1	0.21	EGFL8	0.19	NICN1	0.17	YJEFN3	0.16
**MYH7B**	0.25	PCGF3	0.21	ZBTB40	0.19	BTNL9	0.17	KCNMB3	0.16
**FAM47C**	0.25	NEAT1	0.21	PLIN5	0.19	FAM47E	0.17	POU2AF1	0.16
**GABRR2**	0.25	ITGA7	0.21	KIAA1530	0.19	LOC389634	0.17	UPK3BL	0.16
**GOLGA6L10**	0.25	MYH3	0.21	RAB2B	0.19	NCRNA00182	0.17	ADCY6	0.16
**L3MBTL**	0.24	MAT2A	0.21	KIAA0831	0.19	GCFC1	0.17	CYB5RL	0.16
**LEAP2**	0.24	LOC100128842	0.21	PNN	0.19	KSR2	0.17	OR52N4	0.16
**KIAA1984**	0.24	LUC7L3	0.21	TIA1	0.19	IQCB1	0.17	ZNF157	0.16
**C1orf213**	0.24	NCRNA00115	0.21	C8orf44	0.19	DDX55	0.17	LRDD	0.16
**LY6G5B**	0.24	DOM3Z	0.21	C1orf228	0.19	TTC31	0.17	SLFN13	0.16
**OGT**	0.24	RFPL2	0.21	AP3B2	0.19	PNMA3	0.17	MRPS25	0.16
**PRODH2**	0.24	C1orf63	0.21	SLC25A35	0.19	SCARNA12	0.17	VIP	0.16

**KIAA0907**	0.24	CYP4A11	0.21	ZSCAN23	0.19	PCDHA8	0.17	ZNF471	0.16
**CELF6**	0.24	ZNF354A	0.20	NRBP2	0.19	ZNF449	0.17	TMEM31	0.16
**LOC91316**	0.24	ZNF204P	0.20	TPH1	0.19	GNRH1	0.17	GAS2	0.16
**SDHAP1**	0.24	NTN5	0.20	INE1	0.19	DPPA2	0.17	DPEP3	0.16
**PRAMEF5**	0.24	RBM6	0.20	CARD8	0.19	GAPDHS	0.17	ZDHHC8P1	0.16
**C17orf69**	0.24	FAM186A	0.20	GGT7	0.19	TSSK4	0.17	TMEM151B	0.16
**LOC284900**	0.24	PAN2	0.20	WDR73	0.19	SYT5	0.17	OR1Q1	0.16
**CLK4**	0.24	INTS3	0.20	FLJ43663	0.19	KCNH6	0.17	EXOG	0.16
**ZMAT1**	0.24	SERINC4	0.20	C16orf86	0.19	ENOSF1	0.17	GSDMB	0.16
**NCRNA00174**	0.24	LOC200030	0.20	RUFY3	0.19	LOC100133161	0.17	AMZ2P1	0.16
**MDM4**	0.24	EPM2AIP1	0.20	OFD1	0.19	AGBL3	0.17	KATNAL2	0.16
**DNHD1**	0.24	MAP3K12	0.20	PCDHA9	0.19	KRTAP5-9	0.17	AMY2B	0.16
**RBM46**	0.23	MTMR9L	0.20	SUZ12P	0.19	ANKK1	0.17	OBSCN	0.16
**SLC4A9**	0.23	DDX26B	0.20	POLR2J4	0.19	SLC25A14	0.17	PCDHA1	0.16
**SFRS5**	0.23	LOC400927	0.20	HCG2P7	0.19	ZNF26	0.17	CRELD1	0.16
**LOC440944**	0.23	LYG1	0.20	PPFIA3	0.19	MAMDC4	0.17	NLRP9	0.16
**CCNL1**	0.23	SLC23A1	0.20	SEPSECS	0.19	PCP2	0.17	CEACAM7	0.16

**ZNF346**	0.23	OR10AD1	0.20	FGFR4	0.18	SNHG10	0.17	BCO2	0.16
**GOLGA8B**	0.23	LOC100133331	0.20	ATHL1	0.18	FLJ36777	0.17	CLDN15	0.16
**BCL2L2**	0.23	MAPK8IP3	0.20	LZTR1	0.18	SPRY3	0.17	GK5	0.16
**NCRNA00201**	0.23	SFRS16	0.20	ASPRV1	0.18	ULK3	0.17	NCRNA00171	0.16
**MSH5**	0.23	LOC220729	0.20	FLJ12825	0.18	AFG3L1	0.17	DPRXP4	0.16
**SNORA39**	0.23	CROCCL2	0.20	WASH5P	0.18	USP6	0.17	NFATC4	0.16
**RRN3P1**	0.23	NAPB	0.20	HCN3	0.18	UNC119B	0.17	C14orf182	0.16
**GOLGA2B**	0.23	CCNL2	0.20	RPL23AP64	0.18	ZNF273	0.17	PRSS30P	0.16
**COQ10A**	0.23	RUFY4	0.20	ZNF682	0.18	NPHP3	0.17	SUPT7L	0.16
**HNRNPH1**	0.23	WASH3P	0.20	FAM22F	0.18	PCDHA2	0.17	ANKRD36	0.16
**RAB24**	0.23	ANKZF1	0.20	STAG3L2	0.18	ATF7IP2	0.17	PPP4R1L	0.16
**VAMP1**	0.23	SNRNP70	0.20	RBM44	0.18	ITGA2B	0.17	LOC100271722	0.16
**LRRC36**	0.23	PYY2	0.20	TAF1C	0.18	PLAC8L1	0.17	SLC25A29	0.16
**SDR39U1**	0.23	CCDC84	0.20	SFRS11	0.18	SYCP2	0.17	ZNF321	0.16
**SLC6A16**	0.23	DCST2	0.20	GIGYF1	0.18	CALML6	0.17	GTF2IRD2	0.16
**CDK3**	0.23	PRRT2	0.20	C17orf56	0.18	C2orf67	0.17	MYCBPAP	0.16
**COL6A4P2**	0.23	KGFLP2	0.20	UQCC	0.18	SLC22A9	0.17	EPO	0.16

**NCRNA00105**	0.23	SYNGR1	0.20	PTBP2	0.18	ZNF814	0.17	SNHG1	0.16
**NSUN5P2**	0.23	GOLGA8A	0.20	ERMN	0.18	LOC645676	0.17	LOC340508	0.16
**COLQ**	0.23	CCDC14	0.20	LOC283314	0.18	NCRNA00107	0.17	ZNF717	0.16
**PRSS50**	0.23	C14orf138	0.20	C1orf220	0.18	C1orf113	0.17	OR5K2	0.16
**PABPC1L**	0.23	LRP5L	0.20	VCY	0.18	AGAP4	0.17	MLLT6	0.16
**LOC100128675**	0.23	SALL4	0.20	PMS2L3	0.18	RAB7L1	0.17	RORA	0.16
**SEC31B**	0.23	DOC2A	0.20	PPP1R1A	0.18	AGAP6	0.17	ZNF302	0.16
**TTLL3**	0.23	SNORA8	0.20	NCRNA00085	0.18	PLEKHM1P	0.17	PRICKLE4	0.16
**CENPT**	0.23	C4orf19	0.20	CFLP1	0.18	SLC5A11	0.17	DYNC2LI1	0.16
**ZNF789**	0.23	C6orf201	0.20	GUSBL1	0.18	MGC16384	0.17	LOC730101	0.16
**DDX17**	0.23	CLK1	0.20	TCTE3	0.18	SGK2	0.17	SS18L1	0.16
**PILRB**	0.23	NCRNA00173	0.20	LOC80154	0.18	ZCWPW1	0.17	TMEM136	0.16
**NFATC2IP**	0.23	NKTR	0.20	CCDC45	0.18	TCAP	0.17	PLIN1	0.16
**HSD17B3**	0.23	LOC100130557	0.20	SPACA1	0.18	TTC18	0.17	DUSP26	0.16
**CREBZF**	0.23	CWC25	0.20	STARD5	0.18	RGL2	0.17	DTNB	0.16
**LOC100286844**	0.23	PATL2	0.20	WHAMML1	0.18	LOC90834	0.17	AGBL2	0.16
**ZNF785**	0.23	CLK2	0.20	ANKRD23	0.18	FAM194A	0.17	CT45A1	0.16

**DNM1P35**	0.23	LOC153684	0.20	POU6F1	0.18	TTLL6	0.17	MASP2	0.16
**STAG3L3**	0.23	ZNF337	0.20	MAGEA9B	0.18	KIAA0664P3	0.17	GATM	0.16
**ZNF169**	0.23	KCNAB3	0.20	NPIPL3	0.18	HARS2	0.17	NAA40	0.16
**CROCCL1**	0.22	RBM5	0.20	TNNI3K	0.18	TAZ	0.17	GGA3	0.16
**ZNF546**	0.22	TDRD12	0.20	C10orf68	0.18	ZSCAN21	0.17	FAM13AOS	0.16
**ACCS**	0.22	C1orf175	0.20	U2AF1L4	0.18	SUOX	0.17	MST1P9	0.16
**CRYGS**	0.22	SFRS6	0.20	SLC7A5P2	0.18	AMT	0.17	FAM183B	0.16
**NPIP**	0.22	B3GAT2	0.20	SLFN14	0.18	EP400NL	0.17	FLJ44606	0.16
**FAM156A**	0.22	LOC286367	0.20	SLC17A1	0.18	ACYP1	0.17	PIM2	0.16
**LOC389791**	0.22	SNHG12	0.20	BAT1	0.18	ZNF700	0.17	ZNF684	0.16
**NSUN5P1**	0.22	DENND4B	0.20	PLGLB2	0.18	GHRLOS	0.17	PHC1	0.16
**SCAND2**	0.22	LOC728743	0.20	ZNF219	0.18	TSSK6	0.17	NCRNA00164	0.16
**ENGASE**	0.22	EPOR	0.20	AP1G2	0.18	N4BP2L2	0.17	ZNF30	0.16
**CBX7**	0.22	TMPRSS5	0.20	FAM182B	0.18	TMEM143	0.17	ANGPTL6	0.16
**HERC2P2**	0.22	SLC26A11	0.20	CYP4A22	0.18	LOC283050	0.17	LOC100170939	0.16
**RBM33**	0.22	ZCCHC3	0.20	GUCA1B	0.18	GTF2IRD2P1	0.17	ATXN7L2	0.16
**LOC100129726**	0.22	ZRANB2	0.20	RNPC3	0.18	WDR88	0.17	ZFP3	0.16

**KLHDC1**	0.22	ZNF500	0.20	TBX19	0.18	LOC100132247	0.17	C4orf39	0.16
**CHKB-CPT1B**	0.22	SUGT1L1	0.20	C20orf46	0.18	C1orf77	0.17	CNTD1	0.16
**SSPO**	0.22	LOC619207	0.20	C7orf53	0.18	TUBB8	0.17	PRKD1	0.16
**KIAA1529**	0.22	POLN	0.20	CAMKV	0.18	ZNF137	0.17	ZBED5	0.16
**CG030**	0.22	STAG3L1	0.20	DCAF8	0.18	HDAC6	0.17	APOBEC3F	0.16
**PRPF39**	0.22	TRIM78P	0.19	DCAF4L1	0.18	GABRB1	0.17	ZDHHC23	0.16
**LPIN3**	0.22	RIC3	0.19	DHRS4L1	0.18	LOC285359	0.17	PLK1S1	0.16
**MATN1**	0.22	GSTM2	0.19	SLC17A3	0.18	LHX4	0.17	RXRB	0.16
**GOT1L1**	0.22	ATG16L2	0.19	ARHGAP33	0.18	PRRT1	0.17	AIFM3	0.16
**PTPRVP**	0.22	MYLK4	0.19	KIAA0125	0.18	UCP3	0.17	GPRASP1	0.16
**CC2D2B**	0.22	LAMB2L	0.19	ZNF10	0.18	GGNBP1	0.17	GUSBP3	0.16
**TRIM41**	0.22	MYO15A	0.19	WASH2P	0.18	C11orf61	0.17	TTLL13	0.16
**ZNF692**	0.22	CEMP1	0.19	RRAGB	0.18	MGC16703	0.17	GNB3	0.16
**ANGEL1**	0.22	LOC100144604	0.19	PLA2G6	0.18	LOC728723	0.17	ARHGEF7	0.16
**MTERFD3**	0.22	NFKBID	0.19	TF	0.18	SLC13A4	0.17	NR2C1	0.16
**LOC92973**	0.22	MIR17HG	0.19	WDR6	0.18	LOC221442	0.17	LOC202781	0.16
**SGK494**	0.22	TBC1D3	0.19	C17orf55	0.18	PAQR6	0.17	SCNN1D	0.16

**LOC100132215**	0.22	PRR3	0.19	P2RX1	0.18	PRCD	0.17	RGSL1	0.16
**FGF17**	0.22	LOC202181	0.19	TUBG2	0.18	RAB26	0.17	ELMOD3	0.16
**HSF4**	0.22	CT45A3	0.19	EVL	0.18	SCAMP5	0.17	ZNF611	0.16
**LOC729678**	0.22	LOC285733	0.19	FNBP4	0.18	RBM19	0.17	AACSL	0.16
**FLJ13197**	0.22	NMNAT3	0.19	ZNF740	0.18	C17orf57	0.17	C12orf27	0.16
**SH2B1**	0.22	CPT1B	0.19	C10orf110	0.18	C14orf176	0.17	ATP6V0A1	0.16
**MST1P2**	0.22	LUC7L	0.19	LOC100272217	0.18	CCDC66	0.17	IMPG1	0.16
**FLJ45244**	0.22	SEPT7P2	0.19	FBXO44	0.18	CCDC76	0.17	MTRF1	0.16
**ZBTB25**	0.22	DFNB31	0.19	ZNF764	0.18	KIAA1407	0.16	C3orf22	0.16
**ZNF83**	0.22	KRTAP5-10	0.19	SPDYA	0.18	GYPA	0.16	KIAA1737	0.16
**C20orf165**	0.21	HOOK2	0.19	DNAH8	0.18	ADHFE1	0.16	DPH1	0.16
**ZBTB49**	0.21	ZNF529	0.19	LOC388152	0.18	ZG16	0.16	NPHS1	0.16
**KCTD7**	0.21	FAM153B	0.19	ZNF391	0.18	NCRNA00204B	0.16	C1orf101	0.16
**MST1**	0.21	LOC100128288	0.19	MAN2A2	0.18	RALGPS1	0.16	AMAC1L2	0.16
**FAM113A**	0.21	C8orf77	0.19	MSTN	0.18	ESPNP	0.16	LRIT3	0.16
**PROCA1**	0.21	C9orf45	0.19	TMEM44	0.18	C20orf12	0.16	CCDC155	0.16
**METT11D1**	0.21	STK36	0.19	SNHG11	0.18	LOC100288778	0.16	PFKFB2	0.16

**PPFIA4**	0.21	WDR52	0.19	CATSPER2	0.18	C19orf44	0.16	ECHDC2	0.16
**TMCO6**	0.21	PPIEL	0.19	ZNF862	0.18	PRPF3	0.16	ARSF	0.16
**IL11RA**	0.21	ZFP62	0.19	PHKG2	0.18	PSMA8	0.16	UNKL	0.16
**C5orf45**	0.21	RUNDC2C	0.19	LOC100131193	0.18	MZF1	0.16	GPX5	0.15
**SFRS18**	0.21	CLCN5	0.19	ROBO3	0.18	CCT6P1	0.16	LUZP4	0.15
**DMTF1**	0.21	RG9MTD3	0.19	GIPR	0.18	ARMC2	0.16	RHOT2	0.15
**ALDH8A1**	0.21	FLJ10213	0.19	NBPF9	0.18	RBM25	0.16	ZNF174	0.15
**LOC100130015**	0.21	PGPEP1	0.19	PPOX	0.18	RIPPLY2	0.16	C2orf52	0.15
**CYP4Z1**	0.21	TBC1D3H	0.19	CYP2D7P1	0.18	LOC728264	0.16	RDH16	0.15
**C6orf26**	0.21	TBC1D3B	0.19	C12orf47	0.18	STK19	0.16	ZNF793	0.15
**RNF207**	0.21	FAM166A	0.19	C9orf130	0.18	FBF1	0.16	NNAT	0.15
**TBC1D26**	0.21	ASB16	0.19	PI4KAP2	0.18	TRPV1	0.16	CLINT1	0.15
**ANKS3**	0.21	EZH1	0.19	CDK11A	0.18	LRRC37A	0.16	LOC339788	0.15
**PGBD4**	0.21	LOC100272146	0.19	MYO15B	0.18	RBMXL2	0.16	ANG	0.15
**GPR148**	0.21	C3orf47	0.19	ZNF37B	0.18	FAM19A4	0.16	DBH	0.15
**ESR2**	0.21	BSN	0.19	WDR17	0.18	HAUS5	0.16	FRS3	0.15
**LOC646471**	0.21	GJC3	0.19	LOC100190986	0.18	GNL1	0.16	CCDC144C	0.15

**SLC22A10**	0.21	HPS4	0.19	TTC23L	0.18	C15orf17	0.16	BEX5	0.15
**LRRC39**	0.21	CAPN3	0.19	PABPN1	0.18	C17orf68	0.16	FBXO24	0.15
**MAP3K15**	0.21	C21orf90	0.19	C16orf52	0.18	LOC400027	0.16	ASPDH	0.15
**FAM95B1**	0.21	TTC21A	0.19	C1orf204	0.18	ZNF397OS	0.16	CCDC57	0.15
**DNAH1**	0.21	ADAM11	0.19	FLJ40292	0.18	ZNF418	0.16	THBS3	0.15
**C14orf148**	0.21	C17orf47	0.19	RFXAP	0.18	ITIH4	0.16	NLRP14	0.15
**C17orf86**	0.21	NCRNA00176	0.19	MTCP1	0.18	RBM11	0.16		

Supplementary Table S2. The correlation coefficients between CYP3A43 and its co-down-regulated gene.

**Gene**	**Pearson CC**	**Gene**	**Pearson CC**	**Gene**	**Pearson CC**	**Gene**	**Pearson CC**	**Gene**	**Pearson CC**	**Gene**	**Pearson CC**
FAM83A	-0.20	TYRO3	-0.18	RAB31	-0.18	RALA	-0.17	PABPC3	-0.17	HNRNPF	-0.16
BAG3	-0.19	GLUD1	-0.18	LRRC59	-0.18	FOSL1	-0.17	TNFRSF21	-0.16	FAM89A	-0.16
RGS10	-0.19	C10orf119	-0.18	OGFOD1	-0.17	ACTR1A	-0.17	ATAD1	-0.16	PHLDA2	-0.16
HK1	-0.19	GDI2	-0.18	SHOC2	-0.17	PPA1	-0.17	HIST1H3C	-0.16	FHL2	-0.16
